# DNA Replication Timing Enters the Single-Cell Era

**DOI:** 10.3390/genes10030221

**Published:** 2019-03-15

**Authors:** Ichiro Hiratani, Saori Takahashi

**Affiliations:** Laboratory for Developmental Epigenetics, RIKEN Center for Biosystems Dynamics Research (BDR), Kobe, Hyogo 650-0047, Japan; saori.takahashi@riken.jp

**Keywords:** DNA replication timing, replication domain, mammalian chromosome, 3D genome organization, single-cell Repli-seq (scRepli-seq)

## Abstract

In mammalian cells, DNA replication timing is controlled at the level of megabase (Mb)-sized chromosomal domains and correlates well with transcription, chromatin structure, and three-dimensional (3D) genome organization. Because of these properties, DNA replication timing is an excellent entry point to explore genome regulation at various levels and a variety of studies have been carried out over the years. However, DNA replication timing studies traditionally required at least tens of thousands of cells, and it was unclear whether the replication domains detected by cell population analyses were preserved at the single-cell level. Recently, single-cell DNA replication profiling methods became available, which revealed that the Mb-sized replication domains detected by cell population analyses were actually well preserved in individual cells. In this article, we provide a brief overview of our current knowledge on DNA replication timing regulation in mammals based on cell population studies, outline the findings from single-cell DNA replication profiling, and discuss future directions and challenges.

## 1. Introduction

Over half a century ago when the discovery of the double helical DNA structure immediately invoked the possibility of semi-conservative DNA replication [1], researchers at the time must have naturally been attracted to solving the mechanism of DNA replication [2]. Under such circumstances, temporal control of DNA replication was discovered more or less by chance, by the use of tritium-labeled thymidine for studying DNA replication and chromosome structure [2]. A myriad of subsequent studies have revealed that DNA replication timing correlates well with transcription, chromatin structure, and the spatial arrangement of chromosomal DNA in the cell nucleus [3,4,5,6]. As such, DNA replication provides an excellent forum in which to investigate various levels of genome regulation and their relationship. However, the notion of DNA replication timing has been rather elusive and it has been extremely challenging to elucidate the regulatory mechanisms. As a consequence, its biological significance remains unclear.

As exemplified by the close relationship between semiconservative DNA replication and the DNA double helix, the process of DNA replication closely reflects the underlying DNA structure. Based on this premise, there has always been a sense of hope that the timing of DNA replication might also reflect some important unknown aspects of chromatin structure [3,4]. In addition, DNA replication is an easy-to-measure output either by biochemistry, molecular biology, or microscopy simply because there are only two possible states (i.e., replicated or unreplicated), and a wealth of technologies and protocols are available from studies over the years. The sense of hope and expectation became a reality when high-resolution genome-wide DNA replication timing maps became available and their uncanny relationship to 3D genome organization as assayed by Hi-C (high-throughput chromosome conformation capture) became apparent [7,8,9,10].

Furthermore, the advent of the recently reported single-cell DNA replication sequencing (Repli-seq) technology [11,12] is allowing DNA replication timing research to, at last, enter the single-cell era. Single-cell Repli-seq (scRepli-seq) methods utilize the fact that DNA replication data can be expressed in a binary manner by copy number analysis (i.e., replicated or unreplicated), meaning that they are inherently robust, reproducible, and easy to compare between different laboratories. Moreover, the genome-wide binary data format is in line with the trend of modern biology, which puts a strong emphasis on computation and large-scale comparison of multiple data sets. In this review, we provide an overview of DNA replication timing studies of cell populations carried out mainly in mammalian cells over the years, explain the key findings made by the scRepli-seq method, and also discuss the potential of this powerful methodology and its impact in the coming years.

## 2. Replication Timing and Transcriptional Activity: A Longstanding Relationship

In metazoans in general, chromosomal domains replicated in early and late S-phase correspond to euchromatin and heterochromatin, respectively [6,13,14,15]. The key finding that led to the establishment of this notion was the discovery of the replication timing difference between the active and the inactive X chromosome, with the latter being replicated later than the former [16]. From the late 1960s to the 1970s, various chromosome banding methods including DNA replication banding were devised and eventually all individual chromosomes became identifiable based on the different stripe patterns [17]. Chromosomal regions replicated in the first half of the S-phase corresponded to the Giemsa-light R bands with high GC content and high transcriptional activity, while regions replicated in the latter half of S-phase corresponded to the Giemsa-dark G bands with low GC content and low transcriptional activity [13,17].

Correlation between early replication timing and transcription was confirmed molecularly by analyzing a dozen or so cell-type specific genes in the 1980s, but the confirmation of whether this relationship held true genome-wide had to wait for the emergence of microarrays and their use for analyses of replication timing and transcription. In the early 2000s, genome-wide DNA replication timing analyses were carried out for the first time in budding yeast [18] and then in *Drosophila* cultured cells [19], and the *Drosophila* study by Schübeler et al. verified the correlation between early replication and transcription genome-wide [19]. Thereafter, multiple genome-wide analyses confirmed this correlation in metazoan cells [20,21,22,23].

Interestingly, such a correlation was not observed in budding yeast [18], suggesting that this relationship was acquired at some point during evolution and may have to do with the increased genome size, cell nucleus size, or multi-cellularity [24,25]. Moreover, replication timing regulation in budding yeast is best explained by stochastic rather than deterministic firing of replication origins with different firing efficiency [4,26,27,28,29]. Stochastic firing of origins is also observed in mammalian cells [30,31,32,33]. At the level of the genome, however, there is a defined temporal order of replication during S-phase in mammals [4,34] and cell-to-cell replication timing heterogeneity is limited (discussed later). This discrepancy could be reconciled if we assume that the degree of stochasticity in origin firing observed in mammalian cells is similar to that seen in budding yeast; in mammals, replication timing variability appears relatively small simply because of their long S-phase, whereas in budding yeast, variability is relatively large due to short S-phase. Based on the size, gene density, and relative replication timing heterogeneity at the genome scale, we favor the view that the gene-dense and Mb-sized budding yeast chromosomes are somewhat equivalent to single early replication domains in mammals. On the other hand, the equivalent of gene-poor and late-replicating subnuclear compartments in mammals may not exist in budding yeast [4,25].

## 3. Developmental Regulation of Replication Timing

If replication timing is correlated with transcription, one would predict that replication timing would change coordinately with changes in transcription during development. Genomic regions whose replication timing differ between cell types had been identified by analyzing individual genes in the 1980s [13], but replication timing changes during differentiation was not observed until 2004, when two reports examined the replication timing of several dozens of genes during mouse embryonic stem cell (mESC) differentiation [35,36]. Although the causality remained unclear, replication timing changes correlated well with transcriptional state of genes. The extent of replication timing differences between different cell types was analyzed first by a polymerase chain reaction (PCR)-based microarray analysis of chromosome 22 (720-bp mean probe size) comparing two distinct human cell types [22]. Actually, their replication timing profiles were quite similar, with only about 1% of human chromosome 22 showing differences [22]. In 2008, replication timing analysis was carried out before and after differentiation of mESCs to neural precursor cells using high-resolution whole-genome comparative genomic hybridization (CGH) oligonucleotide microarrays, which led to the finding that changes affected approximately 20% of the mouse genome [7]. Later, using the same oligonucleotide microarrays as in [7], replication timing analyses of 22 cell lines representing 10 distinct stages of early mouse development were performed, which revealed that nearly 50% of the genome were affected [8]. The data resolution obtained from these high-resolution oligonucleotide microarrays was comparable to those from next generation sequencing (NGS) in the subsequent years [12,37,38,39]. Consistent with studies using mouse cells, analyses of several dozen human cell types have revealed that at least 30% of the human genome exhibited replication timing difference among cell types [9,40]. Thus, at most 70% and 50% of the human and mouse genome, respectively, are constitutively-early or constitutively-late replicating, whereas at least 30% and 50% of the human and mouse genome, respectively, may exhibit replication timing differences between cell types. Taken together, it became clear that genomic sequences subject to replication timing changes during development were much more frequent than previously expected.

## 4. Replication Foci and the ~1 Mb Chromatin Domain Model

**Figure 1:**
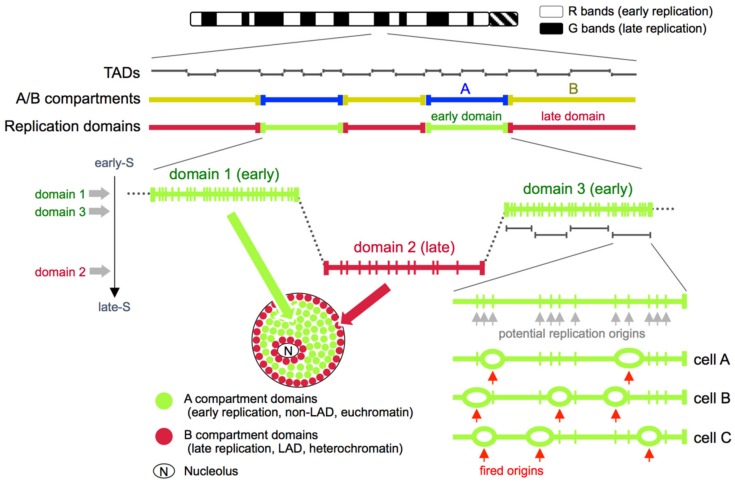
A model for DNA replication timing regulation in mammalian cells. Mammalian chromosomes are comprised of early and late replication domains. Note that in this article we define replication domains as stretches of DNA that show uniform replication timing as defined in [7]. Early replication domains correlate with A compartments (and R bands to a certain extent), whereas late replication domains correlate with B compartments (and G bands to a certain extent) [9,53,54]. Individual replication domains are composed of multiple topologically associating domains (TADs), but in some cases, they may correspond to a single TAD [10]. When two or more adjacent TADs exhibit similar replication timing, they appear as a single replication domain to our eyes. Domain replication timing is determined reproducibly (and nearly deterministically) in early G1-phase at the timing decision point (TDP) [55]. Within a given replication domain, there are many potential replication origins, the density of which is higher in early S-phase [56,57,58]. DNA replication proceeds by the coordinated firing of a subset of these potential replication origins selected stochastically, which results in cell-to-cell domain replication timing heterogeneity [4]. However, the degree of cell-to-cell heterogeneity turned out to be relatively small [11,12]. In the nuclear space, early replication domains occupy the euchromatic and non-LAD A compartment in the interior, whereas late replication domains occupy the perinuclear or perinucleolar B compartment that closely corresponds to LADs [3,4].

The aforementioned genome-wide analyses in mammalian cells provided convincing evidence that DNA replication is regulated at the level of Mb-sized domains, but this notion originally came from DNA fiber autoradiography studies [41,42]. This was later supported by replication banding studies [17] and subsequently by microscopic observations of replicated DNA [42]. That is, since the 1980s a number of groups have carried out microscopic experiments in which replicated sequences were labeled with nucleotide analogs and visualized in the nucleus by immunofluorescence using antibodies specific to these nucleotide analogs [42,43,44,45,46]. As a result, it was concluded that each stretch of DNA replicated within ~60 min could be observed as a discrete bright spot in the nucleus that was named the replication focus. Because these replication foci were stable entities over multiple cell cycles and were regarded to contain ~1 Mb of DNA based on a rough calculation from their fluorescence intensity, a new concept of “~1 Mb chromatin domain” emerged [42,47,48]. In this model, each of these replication foci are regarded as a single stretch of Mb-sized DNA that is folded in 3D to form a structural unit, which is analogous to a multi-replicon structure formed by the near synchronous firing of adjacent replication origins on the linear genome [4,42] (Figure 1). Interestingly, the nuclear distribution of mammalian replication foci changes as the cells progress through S-phase, with the replication foci early in S-phase decorating the euchromatic compartment in the nuclear interior, while those in late S-phase occupy the nucleolar and nuclear periphery [44,45,46] (Figure 1). Although a part of this “~1 Mb chromatin domain” model needs to be revisited based on a recent super-resolution microscopic analysis of replicating DNA [49,50], it is noteworthy that studies of replication foci carried out as early as in the 1980s had already prompted the discussion of the 3D structure and subnuclear positioning of interphase chromosomes [42], which was way before the widespread discussion of this topic in chromatin biology and the emerging field of the 4D nucleome [51,52] in recent years.

## 5. Replication Domains, Topologically Associating Domains (TADs), and A/B Compartments

Due to the emergence of the Hi-C technology [59], advancement in super-resolution microscopy [50,60,61], and the establishment of new disciplines such as the 4D nucleome [51,52] during the last several years, the distance between genome-wide data and microscopic data of chromosome structure is gradually becoming closer [62]. As for replication timing, early and late replicating domains detected by Repli-seq correlate very well with A (euchromatic, active) and B (heterochromatic, inactive) subnuclear compartments, respectively, defined by Hi-C [9,53,54] (Figure 1). It is assumed that the presence of A/B compartments in Hi-C data reflects a state in which topologically associating domains (TADs) [63,64], which are regarded as the basic chromosomal units revealed by Hi-C, are segregated into two mutually exclusive spatial compartments in the nucleus. The mutually exclusive interaction pattern of A vs. B compartment sequences revealed by Hi-C is perfectly consistent with the mutually exclusive spatial pattern of early vs. late replication foci revealed by microscopy (Figure 1). Furthermore, the late-replicating B-compartment sequences also corresponded well with the distribution of the nuclear lamina associated domains (LADs), which show preferential interaction with the inner nuclear membrane [65,66] (Figure 1).

Interestingly, when a collection of DNA replication timing boundaries from 43 genome-wide replication timing data sets representing 18 human cell types were compared to positions of TAD boundaries from human IMR90 fibroblast cells, it was found that roughly 80% of all TAD boundaries coincided with replication timing boundaries [10]. Given that the collection of replication timing boundaries would only increase by analyzing more cell types, it is reasonable to assume that 80% is an underestimate, which led the authors to conclude that there is a near one-to-one relationship between TAD boundaries and a collection of replication timing boundaries of multiple cell types (note that this is actually a collection of “early borders” of replication timing boundaries, to be more specific). According to this view, a stretch of early or late replicating sequence is comprised of multiple TADs, and replication timing boundaries of any given cell type correspond to a subset of TAD boundaries [10] (Figure 1). Moreover, because replication timing boundaries differ between cell types and yet they always coincide with TAD boundaries, it was proposed that TADs could be the regulatory units of replication timing [10]. The exact relationship between these units (TADs) and the ~1 Mb chromatin domains (i.e., replication foci) remains to be explored. However, it is tempting to speculate that these units (TADs) may be identical to the ~1 Mb chromatin domains (i.e., replication foci), as their sizes are reasonably similar (Figure 1). Whether they are identical or not, it is plausible that these units as well as replication domains are not just units of DNA replication (Note that in this article we define replication domains as stretches of DNA that show uniform replication timing separated by timing transition regions, according to the definition in [7]; see also Figure 1). They are also structural units of chromosomes that reflect the chromatin state and subnuclear positioning of sequences throughout the genome.

## 6. Replication Timing and Cell Cycle

DNA replication timing program in mammalian cells is established at the timing decision point (TDP) in early G1 phase, 2–3 h after exit from mitosis [55], although the timing of TDP during G1-phase varies slightly between cell types [67]. In the current view, which of the many potential replication origins within a replication domain will fire is determined in a probabilistic (i.e., stochastic) manner in each cell, in each cell cycle, and on each of the two homologous chromosomes. In contrast, domain replication timing is assumed to be highly reproducible, nearly deterministic, and re-established at the TDP in every G1-phase [4]. This is especially easy to imagine in the case of early replication domains, because they show a high density of potent replication origins, which would minimize cell-to-cell domain replication timing variability regardless of replication origin choice. Interestingly, the TDP corresponds to the time point at which subnuclear positioning of chromosomes [55,68,69] and the global chromatin interaction patterns as assayed by 4C-seq [70] are re-established after exit from mitosis to take on the positioning and interaction patterns that one typically sees in the interphase cell nucleus. It is possible that which subnuclear compartment each domain is allocated to at the TDP will be reflected in its DNA replication timing in the upcoming S-phase. TAD structure, however, is not absolutely necessary to properly sort individual domains into A or B compartments [71,72] or for maintaining proper replication timing profiles [73]. TADs and A/B compartments, while maintaining their overall structure, show interesting changes as cells progress through the S-phase; although the intensity of TAD boundaries decrease upon replication, the intensity of the A/B compartment boundaries gradually increase throughout the S-phase [74].

## 7. Unraveling the Complex Relationship: Replication Timing, 3D Genome Architecture, and Transcription

What is the exact relationship between replication timing, 3D genome architecture, and transcription, which are so complexly intertwined? Elucidating their relationship is a major challenge for the replication timing field, and this endeavor should ultimately lead us to address the molecular mechanism and biological significance of replication timing regulation. So which are separable, and which are inseparable?

One potentially effective means is to observe and compare their kinetics using an experimental system in which all three properties change, such as embryonic stem cell (ESC) differentiation, and look for separability. For example, regarding the relationship between transcription and replication timing during ESC differentiation, not all genes in late-to-early switching replication domains show increase in transcription, and conversely, not all genes in early-to-late switching domains become shut off [7,8,40]. Furthermore, replication timing changes and transcriptional changes were not necessarily coordinated temporally during ESC differentiation: in some cases the former was earlier, but in other cases vice versa was the case, and some genes could be expressed within late-replicating domains in both mice and humans [7,8,40]. Nonetheless, there were many cases in which both changes occurred in a coordinated manner [7,8,9,75], suggesting that they change on a case-by-case basis. Although it has been rather difficult to separate these three properties from each other, replication timing and transcription are separable at least in certain contexts.

Use of relatively unexplored organisms for studying replication is also effective, especially when one focuses on unique developmental stages. Due to the availability and feasibility of NGS, non-mammalian organisms without much information about DNA replication regulation have recently been utilized for genome-wide DNA replication studies, including chicken [76,77], zebrafish [78], *Caenorhabditis elegans* [79], maize [80], *Arabidopsis* [81], *Drosophila* [82] and others [83,84]. In particular, DNA replication profiling during early zebrafish embryogenesis demonstrated the presence of a DNA replication timing program prior to the mid-blastula transition (MBT) [78]. This contradicts with previous reports [85,86], but is understandable given the power and high resolution of NGS data [78]. The pre-MBT replication timing program resembled that of later stage embryos as if it anticipated the initiation of zygotic transcription at the MBT [78], supporting the view that replication timing and transcription are separable.

Perhaps more effective and straightforward, but more challenging means to unravel the complex relationship is to identify factors that regulate DNA replication timing. In yeast, several *trans*-acting factors that strongly influence replication timing have been characterized, such as Taz1 [87] and the Shelterin complex [88] in fission yeast, Rif1 [89,90] in fission and budding yeast, and Fkh1/Fkh2 [91] in budding yeast, which provided evidence for the importance of proper subnuclear compartmentalization or spatial clustering (i.e., 3D genome organization) of certain sets of replication origins [88,92].

In mammals, on the other hand, although the motivation has been high for the discovery of *trans*-acting factors that influence replication timing, it has proven to be a major challenge and the progress has been rather slow [93]. The single exception is Rif1, the genetic disruption of which triggers extensive replication timing changes comparable to large-scale changes observed during mESC differentiation [93,94,95]. Several other *trans*-acting factors that influence replication timing have been found but their effects are either relatively minor or their mechanisms are not fully understood [67,93,96,97,98,99,100]. As the discovery of such *trans*-acting factors gradually increases, their detailed analyses will follow and our understanding of the relationship between the aforementioned three properties should improve accordingly. This has been demonstrated by the accumulating findings on Rif1 function that followed its initial discovery as a major replication timing regulator [101,102,103,104,105,106,107].

One important unanswered question regarding Rif1 function is the precise relationship between its role in regulating the 3D genome organization [103] and its role in counteracting origin activation by Dbf4-dependent kinase (DDK) through recruitment of protein phosphatase 1 to chromatin [101,102,106,107]. Rif1 also has an unexpected role in promoting replication origin licensing in G1-phase [102], which also has to be factored in. In thinking about these problems, it is important to keep in mind that there are many steps that lead to the execution of DNA replication, from origin licensing to initiation of DNA replication [108]. As a consequence, there are many proteins that are only transiently involved in this multi-step process [108]. Thus, in theory there could be many *trans*-acting factors that influence DNA replication timing and many ways in which this is achieved. Although it has been experimentally challenging to identify *trans*-acting factors, this kind of conceptual framework should be effective in thinking about the exact role of each factor once they are identified. For instance, the fact that DNA replication licensing and initiation occurs in a mutually exclusive manner in G1 and S-phase, respectively [108], and that spatial and structural reorganization of mitotic chromosomes is completed during G1-phase before DNA replication [55,68,69,70], are particularly useful in thinking about this problem.

In addition to *trans*-acting factors, *cis*-acting factors that influence DNA replication timing have also been actively pursued in yeast [27]. These include the effect of telomeric sequences in delaying origin firing [109], which is mediated by factors involved in transcriptional/epigenetic silencing of subtelomeres [110,111,112,113] and by the transient repositioning of subtelomeric late origins to the nuclear periphery in early G1 [114]. Likewise, two tandem telomeric repeats on chromosome arms of the fission yeast were essential for origin suppression activity, which was mediated by binding of Taz1, which prevented the loading of an initiation factor, Sld3, on to the origins [87]. Centromere sequences in budding yeast, on the other hand, replicate early and have the potential to advance nearby origin firing timing [84,115], although this was apparently not conserved in chicken cells [77]. Early replication of centromeric heterochromatin in fission yeast [116] is brought about by the active recruitment of Sld3 to early origins by Swi6 (HP1) [117].

In mammals, it has been reported that a sequence with strong origin activity [76], a sequence that tethered histone acetyltransferases [118], or recruitment of strong transcriptional activators [119] could cause an earlier shift in replication timing of otherwise late replicating sequences. Meanwhile, genetic variations at certain human genomic loci can cause replication timing differences between human individuals [120]. There is also a completely different set of *cis*-acting sequences that affect the replication timing of the entire chromosomes in *cis*, which may involve non-coding transcripts [121]. Overall, evidence for the roles of *cis*-acting sequences in regulating replication timing in mammals are still fragmentary and a unified view has not yet emerged. This situation might change, however, as it was recently reported that deletion of a set of *cis* elements within an early-replicating domain in mESCs caused an early-to-late replication timing change coincident with an A-to-B compartment change [73]. These elements were rich in active epigenetic marks indicative of enhancer activity, and were CTCF-independent loop anchors of intra-TAD long-range interactions. Because some set of deletions that greatly reduced transcription of genes within the domain had little effect on replication timing, changes in replication timing and transcriptional activity could be uncoupled [73]. Meanwhile, changes in replication timing and A/B compartments have not been uncoupled [10,73,75] (although they are temporarily uncoupled in G2-phase in the context of DNA re-replication [122]). These “early replication control elements” (ERCEs) could be predicted genome-wide as CTCF-independent loop anchors located away from LADs that bind p300, Oct4, Sox2, and Nanog, and importantly, the predictions were experimentally validated [73].

## 8. Establishment of Genome-Wide Single-Cell DNA Replication Profiling Methods

Replication timing analyses have been mainly performed by immunoprecipitation of bromodeoxyuridine (BrdU)-substituted DNA (BrdU-IP), i.e., Repli-chip (microarrays) and Repli-seq (NGS) technologies [37,39,53,123,124], or copy-number based methods using cell populations [21,38,120,125]. As such, one has to bear in mind that these genome-wide replication timing maps only represent averaged images of tens of thousands of individual cells and that our knowledge on DNA replication regulation is largely based on these averaged maps. Therefore, whether these averaged replication timing maps reflect the actual replication profiles of individual cells and the degree of replication timing heterogeneity between cells remained unexplored. To address these things, it is necessary to perform genome-wide DNA replication analysis at the single-cell level. An earlier attempt to analyze DNA copy number in single human lymphoblastoid cells suggested its feasibility with microarrays [126], and two research groups recently made this possible by NGS [11,12].

**Figure 2:**
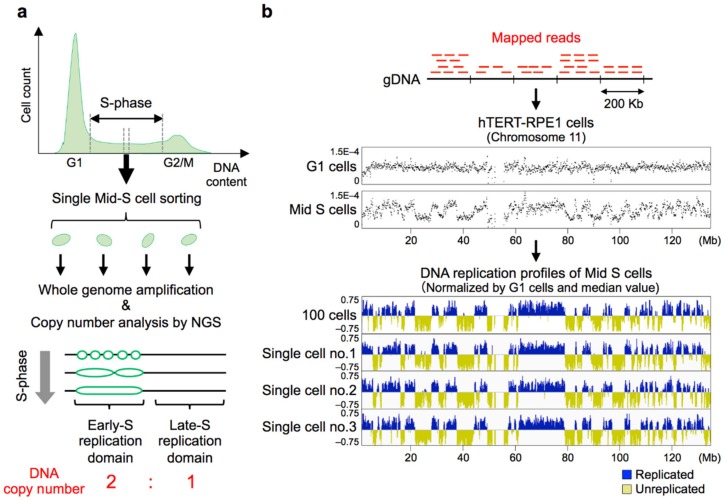
The basic principles of genome-wide single-cell DNA replication profiling (scRepli-seq). (**a**) An experimental overview of scRepli-seq. A typical cell cycle profile of mammalian cells stained with propidium iodide during flow cytometer analysis is shown, along with the mid-S sorting gate used. Genomic DNA samples isolated from single or 100 mid-S cells were subject to copy number analysis by next generation sequencing (NGS) to detect early and late replication domains throughout the genome; (**b**) Replication profiling by copy number analysis. Mapped NGS reads of mid-S cells were counted in sliding windows of 200 Kb (Kilobases) at 40-Kb intervals to generate tag density plots (i.e., counts per window normalized by total read counts). Shown are human chromosome 11 (chr11) tag density data from 100 hTERT-RPE1 cells in G1 and mid S-phase. Mappability was corrected using G1 samples, and the numbers were further divided by the median read count (i.e., median centering) to generate Log_2_[(corrected mid-S)/median] replication profiles of 100 cells and three individual mid-S cells. Figure was adapted from Takahashi et al. [12].

The basic principle of genome-wide single-cell DNA replication profiling is the detection of copy numbers of every genomic sequence in a cell (Figure 2a). In 2018, David Gilbert’s group reported the first genome-wide single-cell replication analysis using haploid/diploid mESCs [11], and our group subsequently reported scRepli-seq profiling of mouse ESCs before and after differentiation, embryonic carcinoma cells, and human TERT-RPE1 (hTERT-RPE1) cells [12]. Both groups focused mainly on mid S-phase cells, in which roughly 50% of the genome have completed replication. Using computational algorithms that can identify copy number differences (i.e., two versus one copy), we could obtain DNA replication profiles from single cells that were remarkably similar to those derived from cell populations (Figure 2b) [12].

Recently, other single-cell epigenome profiling methods are becoming available and epigenetic regulations at the level of nucleotides and nucleosomes such as DNA methylation, histone modifications, and chromatin accessibility can be analyzed in single cells by Bisulfite-seq, ChIP-seq (chromatin immunoprecipitation sequencing), and ATAC-seq (assay for transposase-accessible chromatin sequencing) [127], respectively, although some are still technically challenging and expensive [128,129,130,131]. On the other hand, genome-wide single-cell analyses that address larger-scale chromosome architecture include single-cell Hi-C [74,132,133] and Lamin B1 DamID-seq (DamID: DNA adenine methyltransferase identification) [134], but they are still not commonly used yet. The scRepli-seq technology should be a valuable addition to these single-cell methods that address the 3D chromosome architecture.

Below we will describe the common findings as well as differences between the two reports of single-cell DNA replication profiling.

### 8.1. Replication Profiles Are Stable Among Cells and Are Cell-Type Specific Even at the Single-Cell Level

The replication profiles of individual mid-S cells were found to be very similar to cell population replication timing profiles, in both mice and humans [11,12]. This means that, surprisingly, the same Mb-sized replication domains that we saw in cell population replication profiles were actually present in single cells (Figure 2b). It was also found that cells that represent distinct developmental stages exhibit profiles unique to each cell state [12]. However, the degree of cell-to-cell variability in the two reports was somewhat different even though both groups analyzed undifferentiated mESCs grown in the same condition (i.e., 2i/leukemia inhibitory factor (LIF) medium containing MEK and GSK3 inhibitors (i) and LIF). Actually it was much lower in [12], requiring less than 1.5 h on average to progress from 25% to 75% of cells replicated (defined as T_width_), whereas it was reported that the T_width_ was 2.7 h in [11], assuming a 10-h S-phase. Both groups basically utilized the single-cell copy number profiling protocol developed by Baslan et al. [135], and the overall experimental methods were very similar. This means that small technical differences can have a large impact on the signal-to-noise ratio. We are not completely aware of the source of this variability but there are minor differences in the two protocols such as the method of adding barcodes to individual cells, and it will be necessary to examine these details carefully. In any case, since technical noise will only decrease with future technological advances, it follows that we are currently overestimating the degree of cell-to-cell variability and there is room for discussion as to how much it actually is. Nonetheless, it is clear from the two reports that cell-to-cell variability is fairly low and clustering analysis clearly distinguishes replication profiles of different cell types.

### 8.2. Sources of Cell-to-Cell Replication Timing Heterogeneity

**Figure 3:**
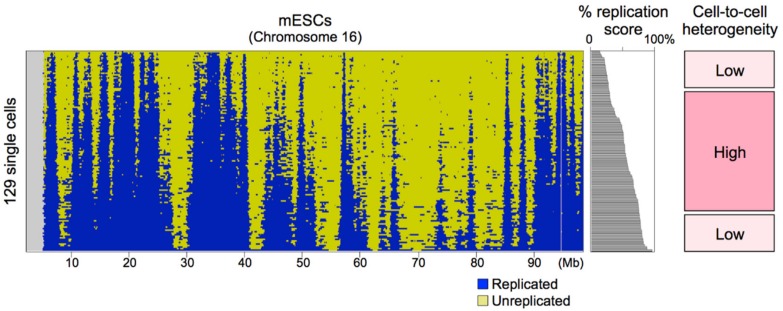
Binarized scRepli-seq profiles of 129 mESCs throughout the S-phase. Data sets were sorted according to the percentage of the genome replicated in each cell. While replication timing heterogeneity was variable among domains, its average was constant and relatively high during the mid S-phase. In contrast, heterogeneity at the beginning and the end of S-phase was much smaller and less variable than mid-S. Figure was adapted from Takahashi et al. [12].

Although the genome-wide replication profiles were conserved among cells, cell-to-cell heterogeneity exists [11,12] and detailed analyses identified genomic regions with relatively high cell-to-cell heterogeneity [12]. One obvious factor was the timing during S-phase. scRepli-seq profiles can be obtained from not just mid-S cells (Figure 2) but also cells throughout the S-phase (Figure 3). When the scRepli-seq profiles of mESCs throughout the S-phase were analyzed, we found three phases of distinct replication timing heterogeneity during S-phase: the degree of heterogeneity was considerably low in early S-phase, relatively high and more variable during mid S-phase, and again considerably low in late S-phase [12] (Figure 3).

For this analysis, 129 cells throughout the S-phase were sorted by the percentage of their genome replicated (i.e., percentage replication score) and we took a sliding-window approach to analyze a total of 19 overlapping 10-percentile groups from the earliest to latest S-phase in increments of 5% [12]. For every genomic bin, the percentage of cells that have replicated the bin was computed for each of the 19 groups and these 19 values were plotted against the average percentage replication score of each group for sigmoid model fitting; the higher the slope of the sigmoid, the less the cell-to-cell heterogeneity. The average values of the slope changed with the progression of S-phase and revealed three distinct phases, leading us to conclude that there are three phases of distinct replication timing heterogeneity [12]. Thus, we believe that our conclusion is not due to analyzing more cells from the middle of the S-phase than early or late S-phase cells.

Replication origin density is highest in regions replicated early in the S-phase, gradually becomes lower and becomes lowest at the end of S-phase [56,57,58]. At the beginning of S-phase, replication timing heterogeneity may be very low simply because of the densely distributed strong replication origins, while genomic regions replicated in the middle of S-phase may show higher heterogeneity because of lower origin density. The very low cell-to-cell heterogeneity at the end of S-phase is counterintuitive given the lowest origin density, and there may be some unknown mechanism that operates to complete S-phase in a timely manner [136,137].

Another source of cell-to-cell heterogeneity identified in mESCs was a set of genomic regions that underwent developmental changes during differentiation [12]. Among them, the regions that changed their replication timing from early to late upon differentiation were found to exhibit significantly higher heterogeneity in mESCs compared to constitutively early and constitutively late regions [12]. This may be related to the observation that genomic regions subject to developmental regulation of replication timing exhibited decreased A/B compartmentalization (i.e., assignment of chromosomal domains to either A or B compartment) compared to constitutively early and constitutively late regions during G1-phase [70]. Together, these observations raise an interesting possibility that the inherent replication timing instability of a subset of developmentally-regulated regions, which probably reflects their compartmentalization instability, may confer competence for developmental regulation.

### 8.3. Haplotype-Resolved Analysis, Allelic Expression Imbalance, and Replication Asynchrony

Single-cell replication sequencing was also compatible with haplotype-resolved analysis using SNPs (single nucleotide polymorphisms)/indels information [120,138], which allowed the profiling of the replication state of single chromosomes in individual cells [11,12]. When the X chromosomes were analyzed in female mESCs, both X chromosomes exhibited similar profiles and had many early-replicating domains. Upon differentiation, however, one of the two X chromosomes clearly replicated late in S-phase [12]. The late replicating state of the inactive X chromosome (Xi) had been observed by genome-wide microarray analysis [8], but haplotype-resolved analysis soon became available to distinguish the Xi from the active X (Xa) [138], and now the distinction between the Xi and the Xa is possible even at the single-cell level. Analysis of autosomes by haplotype-resolved scRepli-seq revealed that replication profiles of pairs of homologous chromosomes are similar overall, suggesting that replication domain organization is stable not only between cells but also between homologous chromosomes.

However, there are some genomic regions that show small but obvious allelic replication timing differences between homologous autosomes. Such regions are evident in the population data of undifferentiated mESCs but these allelic differences tend to disappear upon differentiation [139]. Interestingly, the disappearance of allelic differences was reproducible with scRepli-seq [12]. It is reasonable to assume that the inherent cellular heterogeneity is higher among differentiated mESCs compared to undifferentiated mESCs, especially those grown in 2i/LIF ground-state pluripotency condition [140,141]. Therefore, it is plausible that undifferentiated mESCs have more widespread allelic differences in replication timing than differentiated cells.

When a list of genes that show allelic expression imbalance by population RNA-seq was generated and their replication timing asynchrony was analyzed in single cells, a strong correlation between higher expression and earlier replication was observed [12]. The “uncoordinated” genes (i.e., genes that show higher expression from the later replicating homolog) often had nearby genes that showed the opposite allelic expression imbalance, which could explain the lack of relationship. On the other hand, when a list of chromosomal regions that showed replication asynchrony was generated and the allelic expression state of genes in such domains was analyzed, we often found replication asynchrony without allelic expression imbalance, indicating that the relationship is not one-to-one [12]. However, although the relationship may not be perfect, a coordinated, directional relationship between earlier replication and higher expression clearly exists [12,139]. We speculate that in the context of asynchronous replication and transcription, there may be a possibility that allelic expression imbalance, whether it is direct or indirect (for instance, through A/B compartmentalization), may have an active role in generating asynchronous replication timing. However, it is clear that the relationship between replication and transcription is not a simple one [40] and the relationship is likely to be context dependent. Simultaneous analysis of replication timing and transcription at the single-cell level may provide additional insights in the future.

### 8.4. Single-Cell DNA Replication Profiles Correlate with A/B Compartment Organization

In recent years, it is becoming increasingly desirable to clarify the states and dynamics of the 3D genome organization at the single-cell level [129,130,131], and single-cell Hi-C analyses were reported by multiple groups [74,132,133,142]. However, single-cell Hi-C experiments can, in theory, detect only one DNA-DNA interaction among many possible interactions for a given genomic DNA fragment, making it inevitably difficult to achieve high resolution. Moreover, this limitation allows technical heterogeneity to creep into the data, making interpretation complicated. It should be noted that the field is starting to move past pairwise interactions, as can be seen by the recent development of SPRITE (split-pool recognition of interactions by tag extension) [143], which captures multiple DNA interactions at once without proximity ligation and is conceptually applicable to single cell analysis. At present, however, one cannot identify the positions of TADs and A/B compartments based on single-cell Hi-C data per se.

On the other hand, DNA replication timing profiles very much resemble the A/B compartment distribution, meaning that the A/B compartment organization of individual cells can be estimated from single-cell DNA replication profiles. In fact, Dileep and Gilbert created a Hi-C-like heatmap based on replication profiles derived from single cells, which corresponded well with the actual Hi-C contact map [11]. When assessed quantitatively, mid-S single-cell replication profiles correctly predicted the A/B compartment profiles of more than 80% of the genome [12]. Because this was also the case with haplotype-resolved scRepli-seq data, it was concluded that scRepli-seq profiles could serve as an extremely powerful means to estimate the A/B compartment structure at the level of single cells and single haplotypes. Because single-cell replication profiles were stable from cell to cell, this also implied that the A/B compartment structure might also be conserved from cell to cell [12].

## 9. Future Perspectives of Single-Cell DNA Replication Profiling

### 9.1. Any Cell, Many Cells, Even Heterogeneous Cell Populations

Here, we would like to discuss the potential impact of the scRepli-seq method for future DNA replication timing studies. The scRepli-seq method can be applied to any cell type as long as the cells are proliferating. Since it is a single-cell methodology and flow cytometers are not absolutely essential (discussed later), one can even analyze rare cells that are difficult to obtain in large quantity. As long as proper reference genome information is available, there is no limit to the types of species one can analyze. Importantly, because high read depth is not required to analyze the replication state of the genome at the level of Mb-sized replication domains, the cost per cell is extremely low for a single-cell NGS experiment. For this reason, it is affordable and practical to analyze a large number of cells that are derived from a particular cell population all at once. In fact, it is practically only “DNA-seq” of single cells, which merely requires single cell isolation and whole genome amplification (WGA); there is no need for any additional procedures (such as reverse transcription for RNA-seq). Therefore, it could be the simplest single-cell NGS experiment that one could ever conceive of.

The success rate of the scRepli-seq experiment is very high, and there are some benchmarks for estimating technical noise [12]. Therefore, it is technically possible to objectively exclude “noisy” data sets. In addition, cell-to-cell replication timing variability is relatively small, as discussed earlier [11,12]. That being said, asynchronously replicating cell population includes cells throughout the S-phase, and because of this there is a legitimate concern that it might be difficult to properly classify the replication profiles of individual cells at different time points in S-phase. However, one can retroactively tell the S-phase time point of each cell after scRepli-seq experiments without much difficulty. Therefore, if one can collect a sufficient number of cells evenly from the entire S-phase, a “trajectory” of how cells move through the S-phase should be almost uniquely determined (Miura et al., unpublished observations). Here, a “trajectory” refers to the curved “S-phase segment” that will emerge when displaying multiple scRepli-seq data from various S-phase time points by non-linear dimensionality reduction methods (e.g., t-SNE, or t-distributed stochastic neighbor embedding), with its start and end points being G1 and G2 phase cells, respectively. If technically noisy data can be correctly excluded, cells of the same type or the same differentiation state should together form a unique S-phase trajectory for each cell type. Each cell will be positioned somewhere along the S-phase trajectory of its cell type and the positioning on the trajectory should depend on the S-phase time point of each cell. If a given cell is located outside of a particular S-phase trajectory, it could be concluded that this cell is likely to be another cell type distinct from the cell type of the trajectory.

Thus, even if it is known beforehand that there is heterogeneity in the target cell population, and even if the cells are not synchronized, by securing a certain number of cells it is expected that the cellular complexity, i.e., the number of distinct replication profiles in the cell population, can be deduced. For instance, when we prepared differentiated ESCs and performed their scRepli-seq profiling, we found a few cells that exhibited little replication timing changes during differentiation, and we concluded that these were cells that failed to respond to and differentiate in the differentiation medium [12].

In addition, when examining cells by scRepli-seq during the course of differentiation, the trajectories of DNA replication profiles change as cells differentiate (Miura et al., unpublished observations). In other words, by using the scRepli-seq method, it is possible to analyze the complexity of a cell population from a “3D genome” point of view, which is different from cell profiling methods that are based on single-cell RNA-seq (scRNA-seq). For this reason, the scRepli-seq method should be a very powerful technique to understand the cellular complexity based on 3D genome organization, especially in the context of cancer tissues, cell differentiation, or animal development.

### 9.2. scRepli-Seq in Combination with Copy Number Variation (CNV) Analysis

Because the scRepli-seq method can retroactively tell the S-phase time point of each cell (Figure 3), even flow cytometers are no longer necessary. A small amount of unwanted cells that are outside of S-phase (i.e., G1 or G2/M cells) will be unavoidable, but even those can serve as controls (that show equal representation of all genomic sequences) so they will not be wasted. Therefore, as long as it is an experimental system capable of single-cell isolation, the target of scRepli-seq analysis will be quite diverse. Because scRepli-seq is practically DNA-seq, it is technically possible to combine scRepli-seq with other analyses that measure different cellular properties, which can be related to the scRepli-seq results for their interpretation.

First of all, what seems to be the simplest is a combination of scRepli-seq with karyotype and copy number variation (CNV) analysis. In fact, the feasibility of CNV analysis has already been shown in the two reports [11,12]. Small deletions (i.e., DNA copy number loss) should be detectable even if the data is derived from S-phase cells. The CNV analysis should be of higher standard in systems that allow haplotype-resolved analysis with sufficient read depth. Also, if DNA copy number gain or translocation caused replication timing changes, those sequences might be identified from the single-cell replication profiles. In fact, unnaturally abrupt replication timing changes along the genomic coordinates are signs of translocation, and various chromosomal aberrations have been successfully identified by this method in lymphoblastic leukemia cells [144]. The same strategy could be applied to scRepli-seq data analysis. For instance, it would be very interesting to clarify the relationship between DNA replication timing variability of multiple cell types derived from cancer tissues and their CNV patterns.

### 9.3. scRepli-seq in Combination with scRNA-seq

Combining scRepli-seq experiments with CNV analysis described above does not even require any additional experiment, but combining scRepli-seq with other experiments is certainly imaginable. For instance, combining scRepli-seq with scRNA-seq immediately comes to mind. Basically, it is difficult to extract high quality RNA from fixed cells [145] but the scRepli-seq method does not require cell fixation (Takahashi, unpublished observations). In principle, not only DNA but also high-quality RNA can be collected from the same unfixed cell. DR-seq (gDNA-mRNA sequencing) [146] and G&T-seq (genome and transcriptome sequencing) [147,148] are representative methods that allow simultaneous analysis of DNA and RNA from a single cell, which could be utilized to combine scRepli-seq and scRNA-seq. For instance, the development of an experimental protocol that simultaneously analyzes DNA methylation and transcription from single cells (scNMT-seq) was based on the principles of G&T-seq [149]. Such an example is encouraging and suggests that simultaneous scRepli-seq and scRNA-seq from the same cell is technically feasible. Once this is achieved, then it is anticipated that the precise relationship between RNA-seq profiles and Repli-seq profiles as well as between their cell-to-cell heterogeneity will be revealed at the single-cell level. In addition, one could even attempt to further combine it with Perturb-seq [150], which combines scRNA-seq and pooled genetic perturbation screening by implementing single guide RNA (sgRNA) barcodes that are expressed as mRNAs [150]. Although one has to come up with ways to increase the throughput, this would allow comprehensive screening for replication timing regulators.

### 9.4. Imaging and scRepli-seq

We would like to discuss the ongoing extensive efforts to validate genome-wide data sets with microscopic imaging or vice versa, which is accelerating due to the booming 4D nucleome research, and discuss their relationship to scRepli-seq.

As mentioned earlier, novel structural units of chromosomes at the Mb-scale such as TADs and A/B compartments have become evident thanks to the Hi-C technology, but whether these structural units are conserved at the single-cell level is still a matter of debate. This is mainly due to the limitations in resolution of single-cell Hi-C technologies [74,132,133], but recent technological developments in imaging have enabled the visualization of chromatin domains at the Mb-scale. For example, inactive chromatin domains have been shown to exhibit a more compact structure compared to active domains [151], and TAD or TAD-like structures have been visualized in single cells [152,153,154].

In a latest microscopic chromatin tracing analysis using the Oligopaint technology [154], the authors analyzed the chromatin structure at the Kb scale. When multiple single-cell microscopic data sets were integrated, a chromatin contact map that very much resembled the cell population average Hi-C contact map was obtained: TAD-like structures could be visualized in the target region, and the A/B compartment distribution could also be predicted from the contact map [154]. The contact map derived from single-cell microscopic data sets revealed that the boundary positions of TAD-like structures frequently coincided with CTCF and cohesin-bound sites. However, the coincidence was not 100%, indicating cell-to-cell variability in boundary positioning. Therefore, the extent to which TADs and A/B compartments are preserved at the single-cell level will continue to be discussed in the future.

On the other hand, the A/B compartment organization in single cells can be predicted by scRepli-seq profiles with more than 80% accuracy [11,12]. Because the degree of replication timing heterogeneity among cells was unexpectedly low, it is possible that A/B compartment distribution on the genomic sequences might also be highly conserved among cells [12]. This is in good agreement with the observed spatial segregation of A and B compartment sequences inside the nuclei of single cells by the Oligopaint technology [152], and is also in good agreement with the longstanding observation of the nearly mutually exclusive distribution of early and late replicating sequences in the mammalian nucleus by many laboratories [42].

Although scRepli-seq profiles are an indirect index of A/B compartment organization, they are nevertheless a genome-wide index, unlike microscopic data. Furthermore, a rich body of literature exists on the microscopic observation of DNA replicated at different times in S-phase, which could serve as strong supporting evidence for findings based on scRepli-seq from a microscopic point of view. In the future, it will be interesting to test hypotheses that emerged from scRepli-seq data sets by microscopic analysis. For instance, it will be interesting to visualize the 3D organization of genomic regions that exhibit relatively high cell-to-cell heterogeneity in replication timing and assess their A/B compartmentalization in single cells.

### 9.5. scRepli-seq: Do We Need Higher Resolution?

Although the resolution of the current scRepli-seq method is about 50–100 Kb, which is sufficient to detect Mb-sized replication domains, achieving finer resolution is generally considered preferable and is technically feasible. Based on our own experience and the literature, we predict that the step that affects the data resolution the most is the WGA step [155]. Currently, both groups use a WGA kit based on degenerate oligonucleotide primer (DOP)-PCR called SeqPlex DNA Amplification Kit (Sigma, Darmstadt, Germany), but a novel WGA method called the “linear amplification via transposon insertion” (LIANTI) was developed recently, which is in principle superior to conventional WGA methods and resulted in further reduction of amplification bias [155]. According to the authors, copy number analysis at 10-Kb resolution can be performed without any problem, and the analysis is feasible even at 100-bp resolution. Therefore, LIANTI seems promising and may contribute to improving the resolution of scRepli-seq, which might allow us to describe DNA replication regulation in further detail. The accuracy and resolution of CNV analysis mentioned above would also certainly increase.

However, one should bear in mind that in order to increase data resolution, it will be necessary to increase the NGS read depth, which means more cost. Furthermore, is the current scRepli-seq resolution of 50–100 Kb insufficient in the first place? Is it absolutely necessary to further improve data resolution? Maybe not, because even at the current resolution, important information regarding Mb-scale organization of chromosomes has been obtained [11,12]. In the end, it is the biological question asked that will define the necessary resolution. In light of what we currently know about DNA replication, it could be the stochastic variation in origin firing timing and location [32] that is most likely to be observed by improving the resolution to Kb-scale. If testing whether this is actually the case is an important mission in one’s experimental settings, it would be reasonable to improve the resolution of scRepli-seq. By improving the resolution, we may be able to investigate the degree of coordination between RNA transcription and DNA replication in detail at the single-cell level. In such ways, improving data resolution could lead to new discoveries. However, it should be emphasized that cost-effective and worthwhile discoveries will not be automatically guaranteed if one blindly tries to increase data resolution.

## 10. Conclusions

Genome-wide analyses of single cells are gradually becoming routine and it is now becoming feasible to analyze multiple aspects of the epigenome and the 3D genome architecture by various single-cell methodologies [129,130,131]. Because scRepli-seq is simple, reproducible, and can be applied to any cells that are replicating, it should be a valuable asset to be used by various researchers in the future who want to investigate the Mb-scale organization of the genome through DNA replication. In the future, scRepli-seq, combined with other genome-wide approaches, should lead to a better understanding of the behaviors and states of the genome in single cells in various organs and organisms, as well as to a better understanding of various diseases accompanied by abnormalities in DNA replication and chromosomal structures. Above all, it should serve as a powerful tool to understand the precise relationship between replication timing and 3D genome organization at the single-cell level, which would bring us closer to understanding the biological significance of 3D genome regulation at the Mb scale.
